# Evaluation of the C-lingual Retractor and the Conventional Lingual Orthodontic Brackets in Terms of Speech Performance and Oral Discomfort: A Randomized Controlled Trial

**DOI:** 10.7759/cureus.23752

**Published:** 2022-04-02

**Authors:** Tarek Z. Khattab, Mohammad Y Hajeer, Hassan Farah

**Affiliations:** 1 Department of Orthodontics, Faculty of Dentistry, University of Hama, Hama, SYR; 2 Department of Orthodontics, Faculty of Dentistry, University of Damascus, Damascus, SYR

**Keywords:** oral discomfort, auditive analysis, speech, c-lingual retractor, lingual brackets

## Abstract

Background

The C-lingual retractor (CR) is an alternative lingual technique to retract anterior teeth with minimum torque expression loss. Although the effects of lingual braces upon speech and oral comfort have been studied previously, there is no published data about the C-lingual retractor in this aspect. The aims of this trial were to compare (1) speech performance based on objective acoustic analysis and (2) levels of oral impairment between C-lingual retractor and conventional lingual brackets (LBs).

Materials and methods

A parallel-group randomized controlled trial was conducted on patients with class II division 1 malocclusion who sought orthodontic treatment at the Department of Orthodontics, Hama University Dental School. Thirty-six patients who met inclusion criteria were randomly selected and divided into two groups. Eighteen patients in the C-lingual retractor group (CR group) were treated with a C-lingual retractor, whereas eighteen patients in the lingual brackets group (LB group) were treated with conventional lingual brackets (Stealth H, American Orthodontics, Sheboygan, WI, USA). Fricative /s/ sound spectrograms were analyzed before (T0), immediately after (T1), one month after (T2), and three months after appliance placement (T3). The levels of oral discomfort were assessed using standardized questionnaires to evaluate speech, irritation, chewing difficulties, and other oral impacts.

Results

At all assessment times, the C-lingual retractor caused significant deteriorations in articulation, whereas in the lingual brackets group these deteriorations were statistically significant at T1 and T2 (P<0.001) but not significant at T3 (P=0.073). No intergroup differences were detected. Questionnaire analysis revealed that irritation of the tongue was significantly higher in the lingual brackets group after 24 hours of appliances' placement (P=0.007), whereas speech and mastication problems were insignificantly higher in the C-lingual retractor group.

Conclusions

The findings indicate that the C-lingual retractor has insignificantly a little more interaction with sound production than lingual brackets. Although the levels of oral impacts were almost similar among both groups, more tongue irritation was observed in the lingual brackets group. However, the oral discomfort decreased over the observation period in both groups.

## Introduction

The lingual orthodontic technique, which offers the most esthetic orthodontic treatment option, has developed rapidly [1,2]. However, torque loss during anterior teeth retraction has been considered one of the most challenging problems during lingual orthodontic treatment [3]. The C-lingual retractor mechanics developed by Chung et al. [4] and Kim et al. [5] is an alternative method to achieve a controlled retraction force on the upper anterior teeth [3,6,7]. This technique is a kind of segmental archwire technique that enables the force vector to be moved apically and closer to the center of resistance [7].

Despite its esthetic superiority, lingual brackets cause considerable impairments of speech and other oral functions. Several studies have reported the distortion of articulation after lingual brackets application; some of these studies have employed auditive analysis [8-10], others have assessed speech alteration semi-objectively and/or subjectively [8-12]. The levels of oral discomfort after lingual bracket placement have been investigated as well [8-17]. Although the intensity and duration of oral discomfort and speech difficulties caused by lingual appliances have varied from one study to another, these impairments were well documented in all related papers.

Important concerns regarding the effects of lingual appliance thickness on oral impairments have arisen; some papers have compared the levels of oral discomfort between different lingual appliance systems [12,14], and one paper has tested the effect of lingual bracket size on patients' acceptance [10]. There was a consensus that patients with larger dimensions of the lingual appliance were most commonly affected regarding speech disturbance and oral discomfort.

It seems after reviewing the literature that there is no published data about speech impairments and patients' attitudes after retraction of anterior teeth using lingual appliances. Therefore, we conducted the current study to compare (1) the degree of speech impairment based on auditive analysis and sonography and (2) the levels of oral discomfort between C-lingual retractor and conventional lingual brackets.

## Materials and methods

Study design and setting

A parallel-group randomized controlled trial was conducted at the Department of Orthodontics, University of Hama Dental School, Hama in Syria, between September 2014 and October 2017. The research project was approved by the University of Hama Dental School Local Research Ethics Committee (Reference number: UHDS-169-22032014/SRC-1455) and was funded by the University of Hama Postgraduate Research Budget (ID: UHDS-20157-PG).

Sample size calculation

The following assumptions were used to calculate the required sample size using Minitab®16 (Minitab Inc., State College, PA, USA). (1) The smallest difference requiring detection of the upper boundary frequency (UBF) of /s/ sound was 500 Hz; (2) the significance level of two-sided tests was set at 0.05; (3) the statistical power was set at 95%; (4) the standard deviation (SD) of the boundary frequency of /s/ sound was found to be 404 Hz in a previous study [8]; (5) the intended inferential statistical approach was two-sample t-tests. The calculation revealed that a sample size of 18 patients was required for each group.

Patient recruitment and follow-up

**Figure 1:**
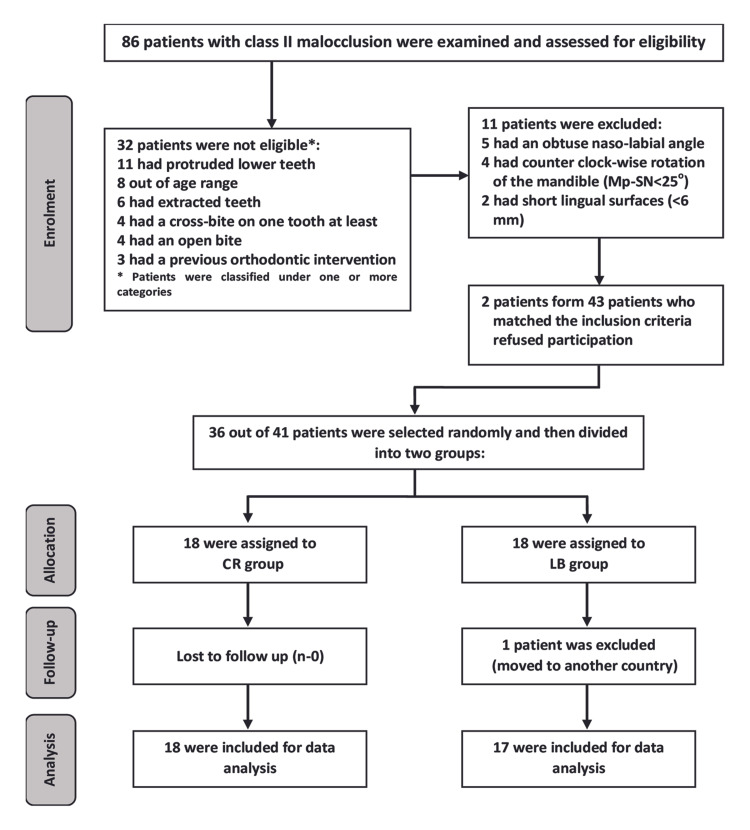
CONSORT flow diagram of patient recruitment, assignment, and follow-up. CONSORT: Consolidated Standards of Reporting Trials. Mp-SN: mandibular plane-anterior cranial base angle.

Participants were derived from patients' records at the Orthodontic Department of the University of Hama Dental School referred between May 2013 and August 2014. A Consolidated Standards of Reporting Trials (CONSORT) flow diagram of participants’ recruitment, follow-up, and entry into data analysis is given in Figure 1. After the clinical, dental cast, and radiographic examination, 43 patients accurately met the following inclusion criteria: (1) class II division 1 malocclusion (according to angle's classification); (2) age range of 17 to 30 years; (3) presence of all permanent teeth, except for third molars; (4) Arabic mother tongue of participants; (5) no craniofacial syndromes, cleft lip and/or palate (soft and/or hard); and (6) no history of speech or hearing disorders. Thirty-six patients were randomly selected and then divided into two groups.

Randomization and allocation concealment

Randomization was performed by one of the academic staff at the Department of Orthodontics (not involved in this research), who created a randomization list with an allocation ratio of 1:1 using Minitab®16. Sequentially numbered opaque and sealed envelopes were used to conceal the allocation sequence from the principal researcher (TZK). Each participant's name and date of birth were written on the envelope, and these data were transferred onto the allocation card inside each envelope. The corresponding envelopes were opened only after all baseline assessments were completed at the time of intervention allocation.

The experimental group: the C-lingual retractor group (CR)

**Figure 2:**
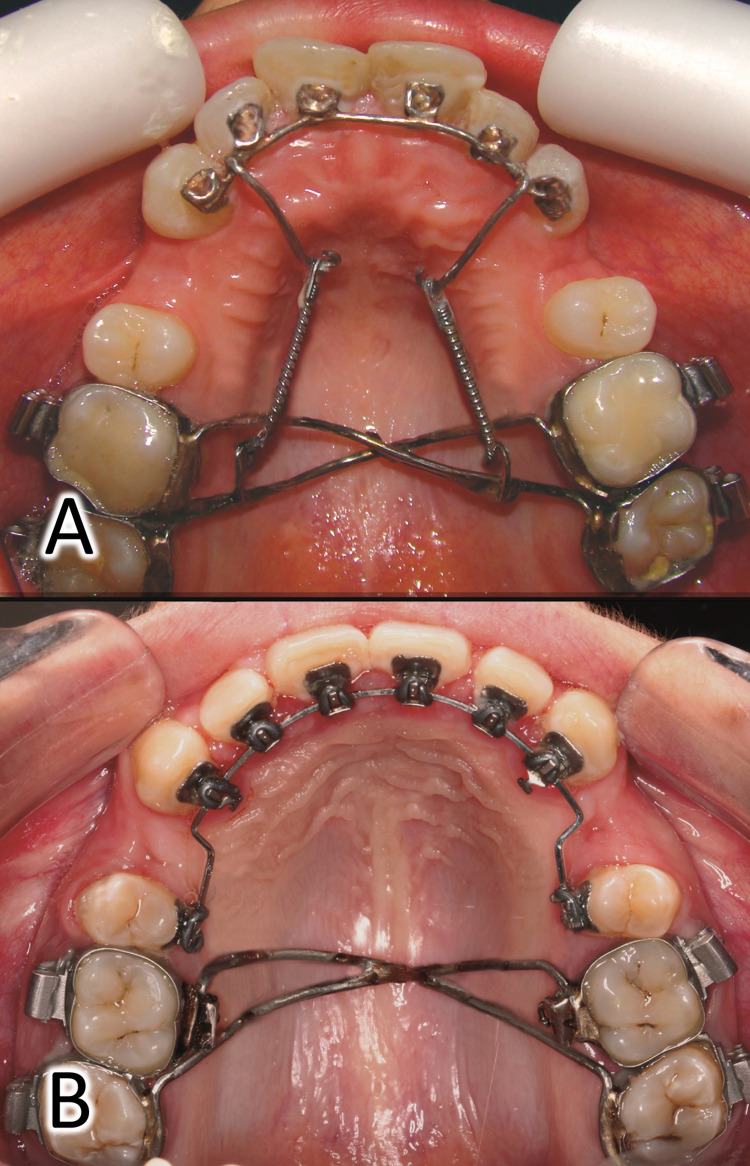
The appliances used in the current trial (A) the C-lingual retractor appliance and (B) the lingual brackets appliance.

This group consisted of 18 patients (10 females and eight males; mean age: 22.3 years; SD: 2.8 years). Patients in this group were treated with a C-lingual retractor appliance (Figure 2). This appliance was made of a 0.9-mm stainless steel wire soldered to pads bonded to the lingual surface of the upper anterior teeth as described previously [4]. Hooks were bent in the wire to change the point of force application so that it passed closer to the center of resistance of the six anterior teeth. The retraction force (approximately 200 g per side) was generated using nickel-titanium closed coil springs (American Orthodontics, Sheboygan, WI). The retraction phase in this group began immediately after appliance placement and before the leveling and alignment stage, which was delayed until the completion of anterior teeth retraction.

The active comparator group: the lingual brackets group (LB)

This group consisted of 17 patients (nine females and eight males; mean age: 23.3 years; SD: 2.4 years). Patients in this group were treated with fixed lingual brackets (Stealth H, American Orthodontics, Sheboygan, WI, USA). Using the Hiro system, lingual brackets were indirectly bonded in the upper arch only [18]. Patients in this group underwent conventional lingual treatment started with leveling and alignment stage (Figure 2).

In both groups, approximately 1 mm of bite opening was achieved after bonding slight bite ramps (Resilience®, Ortho Technology, Tampa, FL, USA) to the occlusal surfaces of the first molars, which allowed slight contact between the incisal edges of at least three lower incisors and the upper appliances. Modified chromosome arches were applied to all patients in both groups as anchorage units. To reinforce anchorage, patients were asked to wear high-pull headgear during the night (350 g per side).

Auditive analysis

**Figure 3:**
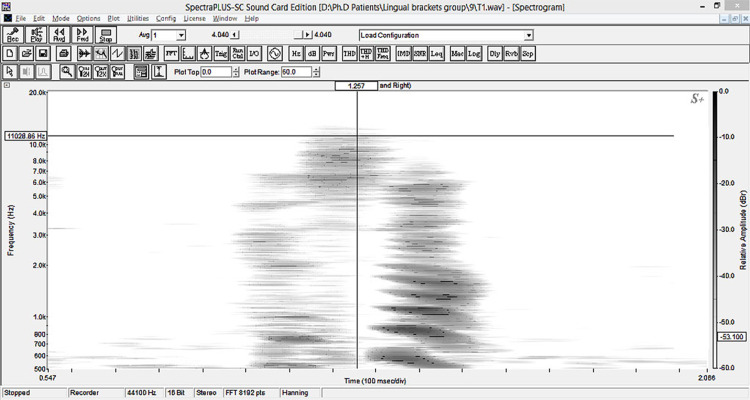
Spectrogram of the Arabic word "Hassan".

Auditive analysis was employed to evaluate speech performance before appliance placement (T0), immediately following appliance placement (T1), one month later (±3 days) (T2), and three months later (±1 week) (T3). Digital sound recording was achieved in these standardized conditions for all patients in all assessment times: (1) each patient was seated in an upright position; (2) recording was done in the same anechoic quiet room; (3) the same microphone was used and placed 10 cm in front of the mentolabial fold. Patients were asked to read the word “Hassan’’ aloud. The target words were then sampled into a computer and the frequency of the /s/ sound was measured using SpectraPLUS® software (SpectraPLUS®-sc, version 5.0.26.21, Pioneer Hill Software LLC, Poulsbo, WA, USA). The upper boundary frequency of the fricative /s/ sound was analyzed using the spectrogram [8-10]. The maximum grayness of the spectrogram represented the maximum frequency of the bandwidth of the fricative sound (Figure 3).

Subjective assessment of patient acceptance and functional impairments

Patients' acceptance and oral functional impairments after appliances' placements were assessed using a standardized questionnaire that consisted of six questions [8,10]. Patients were asked to rate their experienced speech disturbances, oral discomfort, and chewing difficulties on four-point Likert-scale possible answers at four assessment times: before appliance placement (T0), one day following appliance placement (T1), one month later (±3 days) (T2), and three months later (±1 week) (T3).

Blinding

Blinding was applied during data analysis only as the outcome assessor was unaware of the group to which the patient belonged or the administration time for both auditive and questionnaire data.

Statistical analysis

The data were analyzed using Minitab®16 (Minitab Inc, State College, PA, USA). Anderson-Darling normality tests were used to evaluate the distribution of collected data. Intergroup differences were tested using two-sample t-tests or Mann-Whitney U tests when the assumption of normality was violated. Intragroup changes were evaluated using paired-sample t-tests or their nonparametric equivalents, Wilcoxon matched-pairs signed-rank tests, when the assumption of normality was violated. The level of significance was set at 0.05.

## Results

Baseline sample characteristics

The baseline characteristics of the included patients in each group are shown in Table 1.

**Table 1:** Baseline sample characteristics (age and sex). * CR: C-lingual retractor group, ** LB: lingual brackets group.

Variable	CR * (n= 18)	LB ** (n= 17)	Both groups (n= 35)
Age in years (mean ± SD)	22.37 ± 2.8	23.54 ± 2.4	22.82 ± 2.14
Sex: n (%)			
Male	8 (44.5%)	8 (47%)	16 (45.7%)
Female	10 (55.5%)	9 (53%)	19 (54.3%)

Auditive analysis

Immediately after appliances' application at T1, a highly significant drop in the upper boundary frequency (UBF) of the fricative /s/ sound was recorded in both groups (Table 2).

**Table 2:** Speech evaluation before appliance application (T0), immediately following bonding (T1), one month after (T2), and three months post-appliance placement (T3) by auditive analysis of spectrographs. T0: Before the intervention, T1: immediately following bonding, T2: one month after, T3: three months post-appliance placement. ^a^SD indicates standard deviation. *Denotes a significant difference (P<0.05). CR: C-lingual retractor group and LB: lingual brackets group.

Upper boundary frequency (Hz)	CR (n=18)			LB (n=17)			P-value CR vs LB
	Mean	SD^a^	P-value	Mean	SD	P-value	
T0	13,177	411		13,202	506		
T1	10,370	426		10,817	553		
Change T1-T0	−2,808	579	T1 vs T0<0.001	−2,385	844	T1 vs T0 0.001	0.097
T2	12,151	462	T1 vs T2<0.001	12,366	696	T1 vs T2 0.001	
Change T2-T0	−1,026	505	T2 vs T0<0.001	−836	874	T2 vs T0 0.001	0.442
T3	12,828	523	T2 vs T3=0.001*	12,986	510	T2 vs T3 0.008*	
Change T3-T0	−349	591	T3 vs T0=0.023*	−216	465	T3 vs T0 0.073	0.463

Questionnaire findings

At T0, all answers in relation to the six questions were identical (patients chose the answer ‘‘no, not at all”). Therefore, these data are omitted from Table 3.

**Table 3:** Patient responses on the questionnaires were administered at three assessment times following appliance placement in the two groups. T1 indicates one day following appliance placement; T2, one month later; T3, three months later. *Significant difference P<0.05 and NA: not applicable (i.e., identical percentages of frequencies). Question 1: ‘‘Do you feel that your articulation has changed?,’’ Question 2: ‘‘Has a change in your articulation been noticed in your social environment?,’’ Question 3: ‘‘Do you avoid specific types of conversation (e.g., on the phone)?,’’ Question 4: ‘‘Do you notice sores, reddening, or lesions on your tongue?,’’ Question 5: ‘‘Do you have difficulty in chewing?,’’ and Question 6: ‘‘Do you have a sense of your tongue space being restricted?.’’ Possible answers: (1) indicates ‘‘no, not at all,’’ (2) ‘‘slightly,’’ (3) ‘‘yes, to some degree,’’ and (4) ‘‘yes, indeed, I can confirm this.’’

	LB (n=17)					CR (n=18)					P-value, CR vs LB
	Patients' response%					Patients' response %					
	1	2	3	4	P-value (vs T0)	1	2	3	4	P-value (vs T0)	
Q1											
T1	0	27.8	16.7	55.5	<0.001	0	29.4	35.3	35.5	0.001	0.447
T2	38.9	22.2	33.3	5.6	0.004*	11.8	64.7	23.5	0	0.001*	0.778
T3	50	38.9	11.1	0	0.009*	82.3	17.7	0	0	0.181	0.086
Q2											
T1	0	16.7	50	33.3	<0.001	5.9	11.7	47.1	35.3	0.001	0.928
T2	55.6	27.8	16.6	0	0.014*	52.9	35.3	11.8	0	0.014*	0.855
T3	88.9	11.1	0	0	0.371	76.5	23.5	0	0	0.1	0.541
Q3											
T1	44.4	55.6	0	0	0.006*	41.2	52.9	5.9	0	0.006*	0.754
T2	83.3	16.7	0	0	0.181	88.2	11.8	0	0	0.371	0.817
T3	100	0	0	0	NA	100	0	0	0	NA	NA
Q4											
T1	16.7	38.9	33.3	11.1	0.001*	0	5.9	46.7	29.4	0.001	0.007*
T2	77.8	16.6	5.9	0	0.1	41.2	41.2	17.6	0	0.006*	0.062
T3	100	0	0	0	NA	88.2	11.8	0	0	0.371	0.371
Q5											
T1	0	0	27.8	72.2	<0.001	0	5.9	23.5	70.6	0.001	0.882
T2	33.3	16.7	38.9	11.1	0.003*	41.2	35.2	11.8	11.8	0.006*	0.364
T3	50	27.8	22.2	0	0.009*	82.3	11.8	5.9	0	0.181	0.096
Q6											
T1	0	38.9	16.7	44.4	<0.001	5.9	41.2	29.4	23.5	0.001	0.330
T2	44.4	50	5.6	0	0.006*	76.5	23.5	0	0	0.1	0.095
T3	88.9	11.1	0	0	0.371	100	0	0	0	NA	NA

Subjective assessment of speech (Question 1)

At T1, immediately after appliance application, patients in both groups reported a significant deterioration in their voice articulation. One month later, 66.1% of patients in the CR group and 88.2% of patients in the LB group recorded different degrees of speech impairments. After three months of appliance placement, most patients in the LB group (82.3%) and half of the patients in the CR group reported that they were no longer feeling a change in their speech. The differences between the two groups were statistically insignificant at all assessment times (Table 3).

Surrounding people's observation (Question 2)

At the first assessment time, all patients in the CR group and most patients in the LB group (94.1%) stated that the change in their articulation was noticed by their surrounding people. After one month of appliance placement (T2), approximately half of the patients in both groups still rated their articulation as notably restricted. No statistically significant differences were observed between the two groups.

Conversations' avoidance (Question 3)

More than half of patients in both groups reported that they did avoid some types of conversations immediately after appliance application (T1), and the difference between the two groups was insignificant (P=0.754). Most patients after one month (T2) and all patients at the last assessment period (T3) did not avoid any type of conversation in both treatment groups.

Irritation of the tongue (Question 4)

All patients in the LB group and most of them in the CR group (83.3%) suffered from some degree of tongue irritation immediately following appliance placement. The difference between the two groups was statistically significant at this time point (P=0.007). At T2, some patients in the CR group (22.5%) and more than half of patients in the LB group (58.8%) still had a sense of soreness or irritation of the tongue with no significant difference between the two groups (P=0.062; Table 3).

Difficulty in mastication (Question 5)

More than two-thirds of the patients in both groups suffered from severe degrees of eating problems immediately after appliance placement. One month later, an important improvement in patients' assessments of their mastication was observed, but it was still significantly poorer than the records registered before appliance application (at T0). After three months, half the patients in the CR group and most of them in the LB group (82.3%) reported being able to eat comfortably. The intergroup differences were insignificant at all assessment times.

Restriction of tongue space (Question 6)

At T1, all patients in the CR group and the majority of them in the LB group suffered from some degree of tongue space restriction. One month later, a considerable improvement occurred in the LB group when 76.5% of patients had no sense of tongue space restriction and the difference between T2 and T0 in this group was insignificant (P=0.112), whereas this complaint was observed for more than half of the patients in the CR group with a significant difference between T2 and T0 (P=0.006). However, no significant intergroup differences were detected at all assessment times (Table 3).

Harms

No harm or severe untoward effects were observed during the trial.

## Discussion

It seems that the current study is the first to investigate the effects of lingual retraction techniques upon speech performance and oral functional comfort.

Several studies have taken into account the effects of the orthodontic intervention on vowels and voiceless fricative sound formation [19-21]. In particular, the fricative /s/ sound has been widely estimated to evaluate speech performance because this sound, which is common in most languages, is considered to be especially sensitive to morphological changes in the maxillary anterior teeth [8-10]. Moreover, this lingual alveolar sibilant has been considered the most frequently affected sound by dental abnormalities since its production requires precise tongue positioning [22].

A significant drop in the upper boundary frequency (UBF) of the fricative /s/ sound was observed in both groups directly after appliance placement. The C-lingual retractor appliance has the same pathomechanism as the lingual brackets in affecting articulation; the contact area of the tongue is shifted further palatally after placing elements to the lingual surface on the upper anterior teeth [8-10], or even when those teeth are tipped lingually [23]. Laine concluded that patients with a narrower palate showed a tendency of /s/ sound distortion [24]. Similarly, the orthodontic elements that were attached to the lingual surfaces of the teeth may have constricted three-dimensionally the oral cavity and restricted tongue movements during articulation. Patients in the C-lingual retractor group showed more reduction of the UBF than those in the lingual brackets group. This finding can be explained by the difference in the appliances' thickness since the C-lingual retractor has larger dimensions compared to the lingual bracket appliance. Moreover, the C-lingual appliance had elements in different axial levels (meshes were positioned at the level of teeth crowns, and hooks were positioned at the level of the six anterior teeth's center of resistance), whereas the lingual bracket appliance was positioned approximately at the same level (teeth crown level). This difference in the spatial positioning between the two techniques may have played a role in affecting tongue space and thus speech production.

The current sample consisted of class II division 1 malocclusion patients who were treated on a first-premolar extraction basis. A similar sample was evaluated in a previous paper comparing two types of lingual brackets [10]. However, the observation period in the current study was only three months, making our results much more comparable with other published papers dealing with non-extraction cases using lingual fixed appliances and undergoing leveling and alignment procedures. At the third-month assessment time, all of our patients in the conventional retraction group were still in the leveling and alignment stage, whereas those in the C-lingual retractor group were still at the beginning of the retraction stage.

The Arabic word "Hassan" was evaluated previously [8,10], and a similar reduction of the UBF of the /s/ sound was registered when the same lingual brackets that were applied in the current study were used [8], whereas a lower reduction was observed when smaller-dimensions brackets were applied [10]. Less decreased values of the UBF of /s/ the sound were reported by Hohoff et al. [9]. This can be explained by the voice pattern variation when pronouncing different words in different languages [10]. The stressed /s/ sound in the Arabic word “Hassan” was evaluated in the current study, whereas the /s/ sound of the French word “soleil” at the end of the sentence “La brise et le soleil” was evaluated by Hohoff et al.

In the present study, we instructed patients in both groups to wear high-pull headgear during the night to reinforce the anchorage. Recorded pain and discomfort accompanied by headgear wear have varied from one study to another [25-27]. However, the potential influence of these extra-oral appliances on the overall outcomes in our study was considered unlikely because both force magnitude and duration were in their minimal values.

Questionnaire findings revealed that speech impairments were still significantly noticeable three months after C-lingual retractor placement and one month after lingual bracket placement. This can be explained by the difference in the appliances' thickness and the additional components that were incorporated with the C-lingual appliance, such as soldered hooks and close-coil springs. Moreover, patients in this group underwent immediate anterior teeth retraction, which may have caused additional pain and an increase in the levels of general oral discomfort. Hohhof et al. [11] assessed articulation after applying three types of lingual appliances. They concluded that the thinner the appliance was, the less interaction with pronunciation occurred. In our study, a few patients in the lingual brackets group still noticed a mild change in their articulation. These findings agree in general with those of previous studies [8,10,13,15-17,28].

Patients in both groups suffered from some degree of irritation and soreness of the tongue, but significantly higher in the lingual brackets group immediately after appliance placement. This can be explained by the shape of the Stealth® (American Orthodontics, Sheboygan, WI, USA) lingual brackets, which had sharp hooks [8], whereas the C-lingual retractor had relatively smooth-welded surfaces. Another possible reason is the presence of lingual premolar brackets in the lingual brackets group, which did not exist in the C-lingual retractor group. In addition, the mushroom-shaped archwires with the canine offset may have contributed to the tongue irritation in the lingual brackets group. After three months of intervention, only two patients with lingual brackets had a persistent complaint of tongue irritation; similar findings were reported previously [8,29].

Tongue space restriction was noticed by the patients in both groups, and it was insignificantly higher in the C-lingual retractor group. It was confirmed that more intervention with tongue space occurs with thicker lingual appliances [11,12,14]. Haj-Younis et al. [10] investigated the effects of lingual bracket size on tongue space and concluded that the seventh-generation brackets caused more narrowing of tongue space than the STb ones. Another significant impact of the lingual appliances' thickness on tongue space restriction was registered by Hohoff et al. [12] after applying the same lingual appliances with different laboratory positioning techniques. Although the anterior teeth retraction started immediately in patients with the C-lingual retractor in the current study, it seems that this procedure did not have that effect on patients' assessment of their tongue space restriction because the follow-up period was only three months and a relatively small amount of retraction occurred by that time.

Almost the same degrees of mastication difficulty between the two groups were recorded in this study. Many factors may cause chewing difficulty with these interventions, such as irritation of the tongue, posterior bite blocks, and tongue space restriction. After three months of appliance application, mastication ability was insignificantly more often affected in patients with C-lingual retractor appliances than in those with lingual brackets. These findings are in agreement with previous studies that reported similar levels of mastication problems [8,10,12]. Wiechmann et al. [30] reported lower levels of discomfort during eating when they used customized brackets (Incognito, TOP Service, 3M company, St. Paul, Minneapolis, USA), in which brackets were bonded as close as possible to teeth surfaces, producing a thinner lingual appliance when compared with our lingual appliance that was laboratory produced by the Hiro technique. Stamm et al. [14] found that seventh-generation brackets caused significantly more chewing impairments than those caused by customized brackets (Incognito, TOP Service).

The results of our study revealed that chewing difficulty was the most severe problem that met patients with either C-lingual retractors or lingual brackets. These findings agree with previous studies that reported eating problems as the most common problem caused by lingual bracket placement [8,10], but do not concur with those of Fillion [16] and Fritz et al. [15], who found that tongue discomfort was the most severe problem with lingual brackets. Our results also do not agree with the results of Caniklioglu and Ozturk [17] and Wu et al. [13], who reported speech impairments as the most serious problem with lingual appliances.

The generalizability of these results might be limited. In this trial, only patients with class II division 1 malocclusion were involved, and other types of malocclusion were not considered. Therefore, there is a need for more trials with larger samples to evaluate the influence of lingual appliances on speech performance and oral comfort among different types of malocclusion, taking into account the effect of age and gender. Additionally, in the current study, the results of the C-lingual retractor were compared with those of the lingual fixed appliance, in which a specific type of lingual bracket was used. Further investigations using other available lingual brackets are needed. Another source of limitation is that our acoustic analysis was based on one single consonant pronounced by Arabic-speaking patients, and expanded auditive analysis is required in future research work.

## Conclusions

Both appliances caused significant impairments of speech articulation based on auditive analysis and questionnaire-based analysis, but these impairments diminished gradually. Although patients with C-lingual retractor appeared to be more affected regarding chewing difficulties and tongue space restriction, no statistically significant differences were found between the two groups. A significantly more severe tongue irritation was recorded in the lingual brackets group. However, gradual improvements occurred in both groups over the assessment periods.
